# ^1^H MRS-based metabolite changes at ventral respiratory control centers of the medulla oblongata following administration of morphine in wild-type and GIRK2 mutant mice

**DOI:** 10.1016/j.crphys.2025.100147

**Published:** 2025-05-20

**Authors:** Ozra Dehkordi, Stephen Lin, Safia Mohamud, Richard M. Millis, Paul C. Wang

**Affiliations:** a Department of Neurology, Howard University Hospital, Washington, D.C. 20060, United States; b Department of Radiology, Howard University Hospital, Washington, D.C. 20060, United States; c Department of Physiology, American University of Antigua College of Medicine, Antigua and Barbuda; d Department of Physics, Fu Jen Catholic University, Taipei, Taiwan

**Keywords:** ^1^H MRS, Pre-bötzinger, Morphine, GIRK2 channels

## Abstract

Respiratory depression is the leading cause of death in opioid overdose and is closely associated with the development of tolerance following repeated morphine use. However, the neurochemical adaptations in brainstem regions that regulate breathing, particularly under chronic opioid exposure, remain poorly understood. G-protein-gated inwardly rectifying potassium (GIRK) channels, especially the GIRK2 subunit, are expressed in rhythm-generating neurons of the pre-Bötzinger complex and have been implicated in opioid-induced respiratory depression. Nonetheless, their specific role in morphine-induced neurochemical changes is not yet fully defined. In this study, *in vivo* proton magnetic resonance spectroscopy (^1^H MRS) was used in mice to assess morphine-induced metabolite changes in ventral brainstem regions encompassing the pre-Bötzinger complex. Wild-type mice were compared with GIRK2 heterozygous (GIRK2^+^/^−^) mutants. Baseline levels of several metabolites including glutamate (Glu), myo-inositol (Ins), N-acetylaspartate plus N-acetylaspartylglutamate (NAA + NAAG), and glutamate plus glutamine (Glu + Gln) differed significantly between GIRK2^+^/^−^ and wild-type mice. Despite these baseline differences, many of morphine's effects on metabolite levels were similar in the wild-type and GIRK2^+^/^−^ mice. Morphine increased phosphocreatine (PCr) in both genotypes, while total creatine (Cr + PCr) decreased only in the wild-type mice. Glutamine levels increased significantly in both groups. Notably, NAA decreased in wild-type but increased in GIRK2^+^/^−^ mice, whereas NAA + NAAG decreased in both. These findings demonstrate that chronic morphine exposure induces substantial neurochemical changes in brainstem respiratory centers. Although the GIRK2^+^/ ^-^ mutation altered some of the metabolite responses, it does not fully block morphine's effects, highlighting the complexity of opioid-induced adaptations in the respiratory control networks.

## Highlights

• ^1^H MRS measures morphine-induced metabolite changes at pre-Bötzinger complex.
• Morphine alters Gln-Glu cycle metabolites at pre-Bötzinger complex.
• Morphine alters biomarkers for energy metabolism at pre-Bötzinger complex.
• GIRK2 ± mutation does not prevent all of the morphine-induced metabolite changes.

## Graphical abstract

**Figure:**
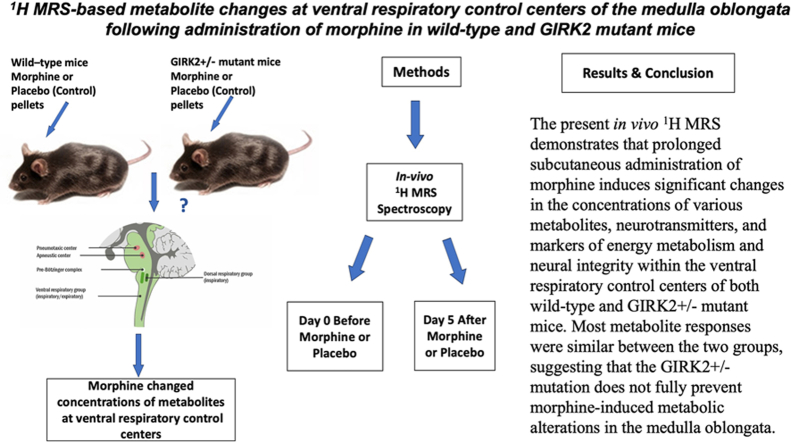

## Teaser

**Significance Statement**: In this study, we used proton magnetic resonance spectroscopy to measure neurochemical changes associated with prolonged subcutaneous administration of morphine in a key respiratory control center in live animals. We compared metabolite changes associated with morphine between normal mice and those with a potassium channel mutation. The results demonstrate that morphine significantly alters the concentrations of several neurochemicals and that the mutation did not fully prevent these metabolite changes. These findings open new avenues for investigating the neurochemical adaptations at brainstem respiratory centers, associated with long-term exposure to morphine.

## 1 Introduction

One of the most widely recognized and potentially fatal acute side effects of opioid analgesics is respiratory depression, and this is of particular concern when opioid drugs are abused [Hall et al., 2008; Pattinson, 2008; Montandon and Horner, 2014; Imam et al., 2020]. However, the exact neurochemical changes associated with opioids at the central respiratory control network are poorly understood. One of the brainstem sites implicated in opioid induced respiratory depression (OIRD) is the pre-Bötzinger complex located among the ventral respiratory group (VRG) in medulla oblongata (Gray et al., 2001; Montandon et al., 2011). μ-opioid receptors that mediate the analgesic and respiratory depressant effects of opioids, are expressed by neurokinin-1 receptor (NK-1R) expressing rhythm generating cells of the pre-Bötzinger complex (Gray et al., 2001; Montandon et al., 2011). μ-opioid receptors are known to modulate neuronal activity through various pathways including calcium channels, adenylate cyclase, and G-protein-gated inwardly-rectifying potassium (GIRK) channels (Montandon et al., 2016; Chan et al., 2017). GIRK channels are expressed by NK-1R neurons of the pre-Bötzinger complex. Studies in GIRK2^−^/^−^ mice have shown that GIRK2 channels expressed by NK-1R cells of pre-Bötzinger complex contribute to a substantial part of the respiratory rate depression following systemic administration of opioids (Montandon et al., 2016). NK-1R stimulation of neuronal activity at the pre-Bötzinger complex has also been reported to occur via inhibition of GIRK2 channels (Montandon et al., 2016). However, the neurometabolite changes associated with opioid activation of μ-opioid receptors at these ventral respiratory control sites are not known and the contribution of GIRK2 channels in the opioid-induced biochemical changes is not clear.

To address these gaps, we employed *in vivo* proton magnetic resonance spectroscopy (^1^H MRS), a non-invasive technique that allows for the real-time assessment of neurochemical changes in the living brain. Unlike post-mortem analyses, ^1^H MRS preserves the physiological environment, maintaining factors such as pH, temperature, and blood flow, and avoids confounding factors related to metabolic degradation or ischemia-induced artifacts [Siesjö, 1978; Tkác et al., 2004; Baranovicova et al., 2023]. This makes ^1^H MRS superior to other methods for quantifying the changes in metabolites associated with prolonged administration of morphine at the brainstem respiratory centers.

Previous ^1^HMRS studies have shown that chronic treatment with morphine modulates concentrations of metabolites such as glutamate (Glu), glutamine (Gln), glutamate + glutamine (Glu + Gln), myo-inositol (Ins), ϒ-aminobutyric acid (GABA) and N-acetyl aspartate (NAA) in different brain regions (Haselhorst et al., 2002; Xiang et al., 2006; Yücel et al., 2007; Hu et al., 2012; Hansen et al., 2014; Dehkordi et al., 2025). In the present study in mice, we hypothesize that metabolite changes induced by prolonged subcutaneous administration of morphine at the VRG are due to activation of the GIRK2 channels expressed by NK-1R cells of the pre-Bötzinger complex. We tested our hypotheses by applying *in vivo* ^1^H MRS to measure the metabolite changes at the VRG associated with subcutaneous administration of morphine to wild-type and GIRK2 heterozygous (GIRK2^+^/ ^−^) mutant mice.

## 2 Methods and materials

### 2.1 Animals

Wild-type and GIRK2 heterozygous (GIRK2^+^/ ^−)^ mice (male and female, 5–6 weeks old) were obtained from the Jackson Laboratory (Bar Harbor ME, USA). The GIRK2^+^/ ^-^ mice were generated via custom embryo cryorecovery of the B6CBACa Aw-J/A-Kcnj6^wv/J (weaver, Stock #000247) line. Recovered embryos were bred with B6CBAF1/J mice (Stock #100011) to establish a stable breeding colony consisting of wild-type and heterozygous GIRK2^+^/ ^-^ mixed-sex mice, specifically for this study.

A total of 32 mice, 16 wild-type and 16 GIRK2^+^/ ^-^ mice, were randomly divided into two treatment groups (n = 8 per group) to receive either morphine or placebo pellets. All procedures, including anesthesia and surgery, were approved by the Institutional Animal Care and Use Committee (IACUC) of Howard University. All efforts were made to minimize the number of animals used and their suffering.

To reduce the nonspecific effects of handling and experimental environment, animals were handled and exposed to the same environment as those used during the actual experiment for a total of 3 days (Handling Day 1–3). On Day 4, animals were anesthetized (isoflurane in 0.8 L/min oxygen at 2–3 % for induction,1–2 % for maintenance) and underwent basal (pre-exposure)

^1^H MRS. On Day 5, mice underwent subcutaneous implantation of morphine pellets (75 mg) or placebo pellets (NIDA, Bethesda, MD, USA) according to previously described protocols (Gold et al., 1994; McLane et al., 2017). This dose of morphine, when implanted under the skin, is reported to result in plasma morphine concentrations sufficient to elicit significant respiratory depression and antinociceptive effects, as evidenced by increased tail-flick latency (Patrick et al., 1975; Hill et al., 2016). For pellet implantation, animals were anesthetized with 2.5 % isoflurane before shaving their hair from the base of the neck. The skin was cleansed with 10 % povidone iodine and rinsed with 70 % ethanol before making a 1 cm incision at the cleansed area. The subcutaneous space was opened, and the pellets were inserted in the space. The site was closed by GLUture topical tissue adhersive (Zoetic Inc. Kalamazoo, MI USA). The animals were allowed to recover in their home cages. Five days after implantation of pellets, the animals were anesthetized and underwent a second ^1^H MRS and immediately thereafter, animals were euthanized.

### 2.2 ^1^H MRS

Animals were anesthetized using isoflurane in 0.8 L/min oxygen at 2–3 % for induction, 1–2 % for maintenance. An animal monitoring unit (SA Instruments, Stony Brook, NY USA) was used to monitor respiration during imaging and spectroscopy acquisition. Depth of anesthesia was assessed throughout the study by monitoring the respiration rate, with target respiration range between 40 and 80 rpm during scanning. A 9.4T Bruker AVANCE 89 mm bore MRI machine was used with a 25 mm RF volume coil. A set of T1-weighted (TE = 8.4ms, TR = 800ms) and T2-weighted (TE = 33ms, TR = 2500ms) spin echo pilot images were acquired in an orientation matching a stereotactic brain atlas (Paxinos and Franklin, 1997) to identify key landmarks for positioning the region of interest (ROI) for spectroscopy. A fieldmap-based localized shimming was applied over the ROI followed by iterative first order shimming, nominally achieving a water peak with <15Hz full-width half-max linewidth. Localized ^1^H MRS was acquired with a point resolved spectroscopy (PRESS) sequence (TE = 15ms, TR = 2.5s, 1024 averages) with variable power and optimized relaxation delays (VAPOR) and water suppression from the pre-Bötzinger complex region. The acquisition was repeated to generate 4 sets of data to be combined in post-analysis. The ROI size for the VRG containing the pre-Bötzinger complex was 1.2 x 1 × 1 mm^3^. The ROI was identified from the mouse brain atlas (Bregma −7.55 mm to −6.59 mm) and matched with MRI images. The ROI (ventral to the nucleus ambiguus and at the level of the inferior olive) was placed in the right brainstem using the morphology of lobule 6 of the cerebellar vermis and the 4th ventricle as guides (Paxinos and Franklin, 1997). In the axial view, the ROI was centered approximately halfway between the midline and the lateral edge of the medulla.

### 2.3 Data processing

Each of the 4 spectra were examined and poor-quality spectra, such as those due to motion, were excluded. The free induction decay (FID) of each spectra were then pre-processed with LCModel's (Provencher, 1993) preprocessor and then summed using a custom MATLAB (MathWorks, Natick, MA USA) script. The resulting summed FIDs were processed using LCModel software to obtain the absolute concentrations of neurotransmitters and metabolites in VRG of the brainstem. LCModel uses a linear combination of model spectra acquired from in-vitro solutions to fit the acquired spectra. A basis set of these model spectra matching the field strength and acquisition parameters of the study (TE = 15ms), was generated and provided by Dr. Provencher. Using an unsuppressed reference water signal, LCModel is able to estimate the absolute concentration with Cramér-Rao Lower Bounds (CRLB), expressed as percentage standard deviation (%SD) of each metabolite. Results with a %SD less than 20 % were deemed acceptable for inclusion in the statistical analysis. The neurotransmitters and metabolites which met the criteria for inclusion were as follows: Creatine (Cr), phosphocreatine (PCr), glutamine (Gln), glutamate (Glu), glutathione (Gsh), myo-inositol (Ins), taurine (Tau), N-acetylaspartate (NAA), NAA + N-acetylaspartylglutamate (NAA + NAAG), glycerophosphocholine + phosphocholine (GPC + PCh), Cr + PCr, and Gln + Glu. All data were expressed as weighted mean ± standard deviation (SD), weighted by %SD.

Two-tailed t-tests were performed to compare baseline metabolite levels in the VRG between wild-type and GIRK2^+^/^−^ mutant mice, as well as to evaluate changes in metabolite concentrations following subcutaneous administration of either placebo or morphine. A p-value of ≤0.05 was considered statistically significant. Changes in weighted metabolite concentrations from day 0 to day 5 were calculated for both treatment groups (placebo and morphine) in both wild-type and GIRK2^+^/^−^ mutant mice. Treatment-related differences were assessed by two-tailed *t*-test for independent samples using standard error of the mean (SEM). SEM was calculated from the standard deviation of the weighted mean concentrations before and after treatment.

## 3 Results

**Fig. 1:**
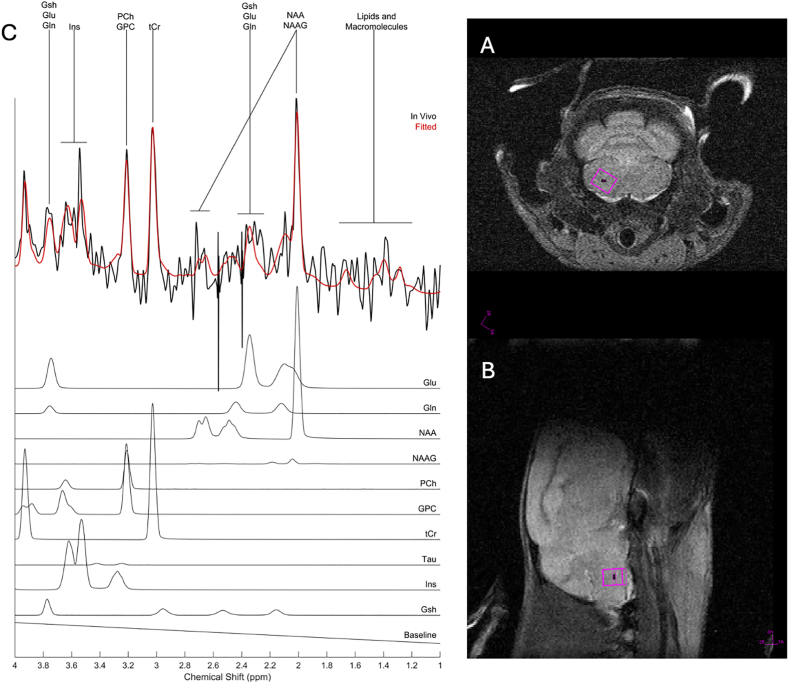
^1^H Magnetic resonance spectroscopy (^1^H MRS) at the ventral respiratory group (VRG). Representative in-vivo ^1^H MRS obtained from the VRG of a control mouse. Panels A and B: Axial and sagittal pilot images, respectively, highlighting the VRG region of interest (size 1.2 x 1 × 1 mm^3^.). Panel C: Typical spectrum from the VRG region of interest in panels A and B with fitted spectrum from LCModel (red) and fitted metabolite subspectra. Relative levels of neurological biochemicals such as glutamate (Glu), glutamine (Gln), N-acetylaspartate (NAA), N-acetylaspartylglutamate (NAAG), phosphocholine (PCh), glycerophosphocholine (GPC), total creatine (tCr), taurine (Tau), inositol (Ins) and glutathione (Gsh) can be identified in a naïve animal.

Fig. 1 shows a representative *in-vivo* ^1^H MRS recording from the VRG of a control mouse. Neurometabolite profiles composed of multiple neurotransmitters and metabolites and their combinations were quantified.

### 3.1 Comparison of baseline metabolite levels between wild-type and GIRK2^+^/^−^ mutant mice

**Fig. 2:**
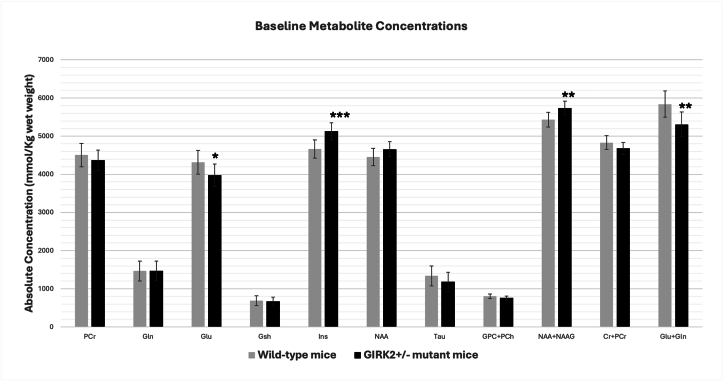
Baseline metabolite differences in the ventral respiratory group (VRG) between wild-type and GIRK2^+^/^−^ mutant mice. Metabolite concentrations were quantified from the VRG on day 0, prior to subcutaneous morphine administration. Data are presented as mean ± SD. Significance levels: ∗P ≤ 0.05, ∗∗P < 0.01. ∗∗∗P < 0.001.

At baseline (day 0), concentrations of some metabolites such as PCr, Gsh, NAA, Tau, GPC + PCh, and Cr + PCr did not differ significantly between wild-type and GIRK2^+^/^−^ mice. However, several other metabolites exhibited notable differences. Specifically, levels of Glu, Ins, NAA + NAAG, and the combined Glu + Gln were significantly different in GIRK2^+^/^−^ mice compared to wild-type controls (Fig. 2).

### 3.2 Metabolite changes in VRG of wild-type mice after morphine treatment

**Fig. 3:**
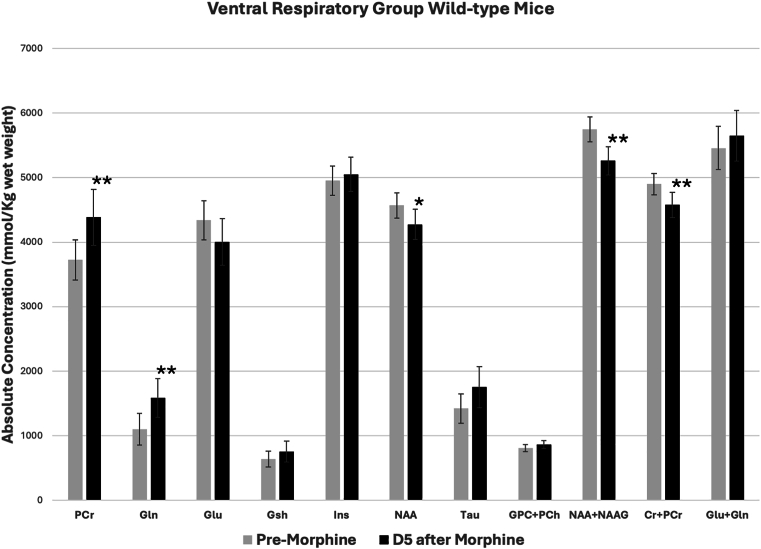
Morphine-induced metabolite changes at the ventral respiratory group (VRG) of wild-type mice. Metabolite concentrations were quantified from the VRG of mice before and 5 days after subcutaneous administration of morphine. Data are presented as mean ± SD. Significance levels: ∗P ≤ 0.05, ∗∗P < 0.01."

Subcutaneous administration of morphine produced significant changes in the concentrations of a number of metabolites in the ventral respiratory neuronal network containing the pre-Bötzinger complex (Fig. 3). PCr, known to play a vital role in cellular energy buffering and energy transport, increased after morphine. Gln component of the Gln-Glu-GABA cycle also increased. Concentrations of NAA, NAA + NAAG and Cr + PCr decreased, whereas Glu, Gsh, Tau, Ins, GPC + PCh and Glu + Gln did not change significantly after morphine.

**Fig. 4:**
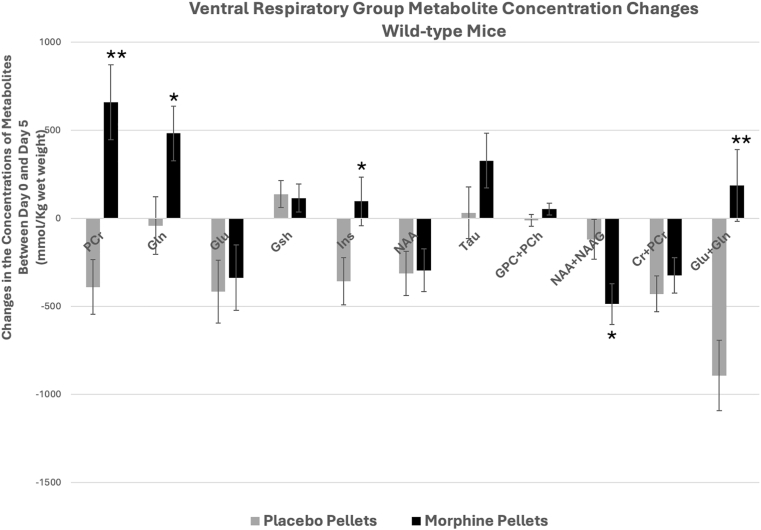
Comparison of placebo- and morphine-induced metabolite changes at the ventral respiratory group (VRG) of wild-type mice. Changes in the concentrations of metabolites between day 0 (no treatment) and day 5 of placebo- and morphine-treated mice. Data represents mean concentration differences for each metabolite (day 0 - day 5) ± SE. Significance level: ∗P < 0.05, ∗∗P < 0.01.

Placebo treatment of the wild-type animals also produced significant changes in the concentrations of several metabolites. Comparison of the changes in the concentration of metabolites between day 0 and day 5 of the placebo and morphine treated animals showed significant differences between the two groups with respect to either the direction and/or the magnitude of changes in PCr, Gln, Ins, NAA + NAAG, and Gln + Glu (Fig. 4).

### 3.3 Metabolite changes in VRG of GIRK2^+^/ ^-^ mutant mice after morphine treatment

**Fig. 5:**
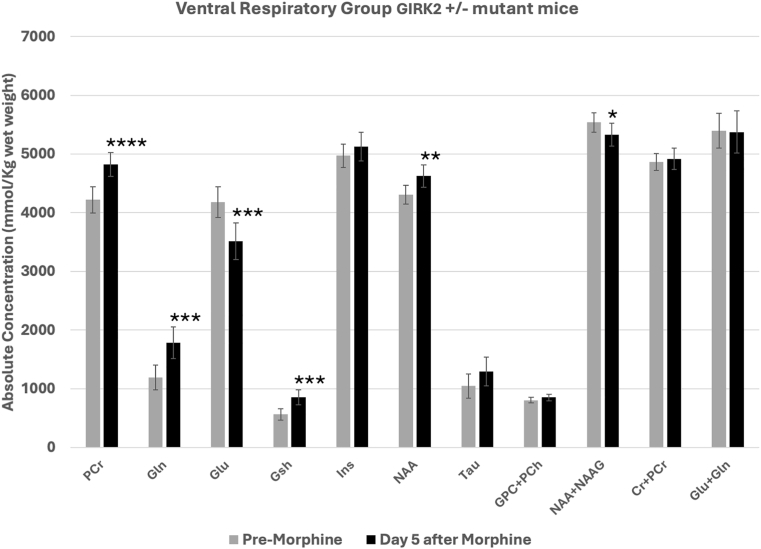
Morphine-induced metabolite changes at the ventral respiratory group (VRG) of GIRK2^+^/ ^-^ mutant mice. Metabolite concentrations quantified from VRG of mice before (Day 0) and 5 days after subcutaneous administration of morphine. Data represents mean ± SD. Significance level: ∗P < 0.05, ∗∗P < 0.01, ∗∗∗P < 0.001, ∗∗∗∗P ≤ 0.0001.

**Fig. 6:**
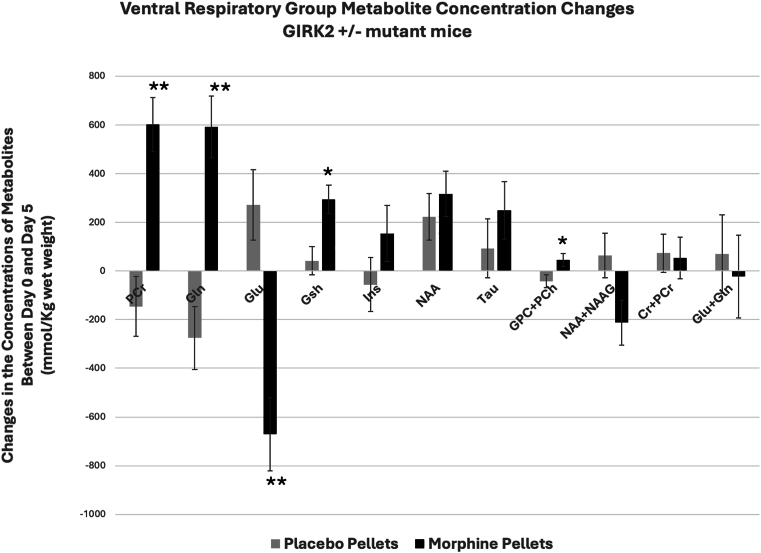
Comparison of placebo- and morphine-induced metabolite changes at the ventral respiratory group (VRG) of GIRK2^+^/ ^-^ mutant mice. Changes in the concentrations of metabolites between day 0 (no treatment) and day 5 of placebo- and morphine-treated mice. Data represents mean concentration differences for each metabolite (day 0 - day 5) ± SE. Significance level: ∗P < 0.05, ∗∗P < 0.001.

In the GIRK2^+^/ ^-^ mutant mice, morphine also produced significant changes in the concentrations of a number of metabolites at the ventral brainstem regions overlapping the pre-Bötzinger complex (Fig. 5). PCr, Gln, Gsh, and NAA increased significantly after morphine. Glu and NAA + NAAG decreased but concentrations of other metabolites such as Ins, Tau, GPC + PCh, Cr + PCr and Gln + Glu did not change. Comparison of the changes in the concentrations of metabolites between day 0 and day 5 of the placebo and morphine treated GIRK2^+^/ ^-^ mutant mice showed significant differences between the two groups with respect to either the direction and/or the magnitude of changes in PCr, Gln, Glu, Gsh and GPC + PCh (Fig. 6).

### 3.4 Comparison of morphine-induced metabolite changes in wild and GIRK2^+^/ ^-^ mutant mice

**Fig. 7:**
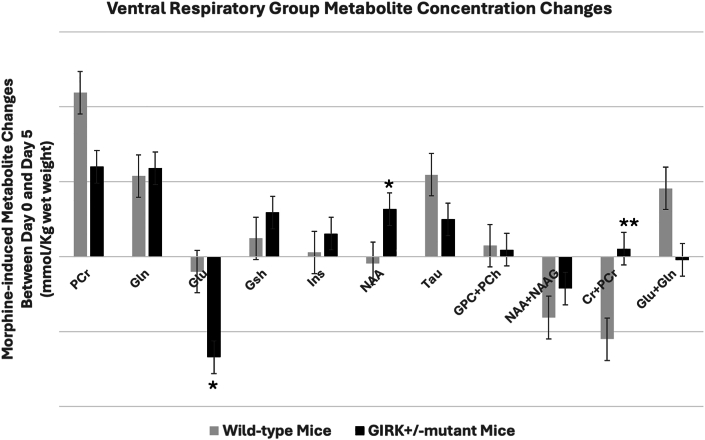
Comparison of morphine-induced metabolite changes at the ventral respiratory group (VRG) of wild-type and GIRK2^+^/ ^-^ mutant mice. Changes in the concentrations of metabolites between day 0 (no treatment) and day 5 of morphine treated wild-type and GIRK2^+^/ ^-^ mutant mice. Data represents mean concentration differences for each metabolite (day 0 - day 5) ± SE. Significance level: ∗P ≤ 0.05, ∗∗P < 0.001.

Comparison of morphine induced metabolite changes in VRG between day 0 and day 5 of wild-type and GIRK2^+^/ ^-^ mutant mice demonstrated a significant difference between the two groups with respect to either the magnitude and/or direction of changes in Glu, NAA and Cr + PCr. However, changes in the concentrations of the other metabolites were not significantly different between the two groups (Fig. 7).

## 4 Discussion

This *in vivo* ^1^H MRS study in mice demonstrates that prolonged subcutaneous administration of morphine induces significant alterations in the concentrations of multiple metabolites in the VRG of both wild-type and GIRK2^+^/^−^ mutant mice. Although most metabolite levels on day 0 did not differ significantly between the two groups, levels of Glu, Ins, NAA + NAAG, and Glu + Gln were notably different in GIRK2^+^/^−^ mutants compared to wild-type controls, suggesting that the heterozygous GIRK2 mutation affects baseline concentrations of certain metabolites. Despite these baseline differences, morphine-induced changes in metabolite concentrations were largely similar between the wild-type and the mutant mice. This opioid responsiveness, even in the presence of partial GIRK2 channel mutation, may reflect residual GIRK2 activity (Signorini et al., 1997). Alternatively, GIRK2 channels may not play a primary role in mediating morphine-induced metabolite changes in this brain region. In support of this idea, previous studies on medullary slices containing the pre-Bötzinger complex, and in awake mice, have shown that μ-opioid receptor-mediated GIRK activation contributed only modestly to OIRD (Wei and Ramirez, 2019). Other studies report that KCNQ potassium channels, independent of μ-opioid receptor signaling, are implicated in OIRD (Abbott, 2020). These interpretations are based on results demonstrating that KCNQ channel activators mimic OIRD and KCNQ-specific antagonists block it (Wei and Ramirez, 2019). In contrast, a GIRK-specific channel blocker failed to reverse μ-opioid receptor agonist-induced OIRD and a GIRK channel activator failed to mimic OIRD (Wei and Ramirez, 2019).

PCr, which serves as a high-energy phosphate donor for ATP formation (Guimarães-Ferreira, 2014; Bresnen and Duong, 2015), increased after morphine in both the wild-type and the GIRK2^+^/ ^-^ mutant mice. This increase in energy reserve at pre-Bötzinger is consistent with morphine-induced respiratory depression (Hall et al., 2008; Pattinson, 2008; Montandonet al., 2014; Imam et al., 2020) which is thought to be associated with a decrease in energy demand.

Gln, the precursor for glutamate, aspartate and GABA, significantly increased after morphine in VRG of both the wild-type and GIRK2^+^/ ^-^ mutant mice. The magnitude of the changes in the concentrations of this metabolite between day 0 and day 5 was also similar in both groups, suggesting that this GIRK2^+^/ ^-^ mutation had minimal effect on the morphine-induced Gln changes. The Glu component of the Gln-Glu cycle, known to be essential for respiratory rhythm generation (Cook-Snyder et al., 2019), decreased after morphine in both groups of mice; however, the decrease in the wild-type did not reach statistical significance. This morphine-induced decrease in Glu implies that the GIRK2^+^/ ^-^ mutation did not block the effect of morphine on this main excitatory neurotransmitter at the VRG containing the pre-Bötzinger complex. Our findings regarding the morphine-induced decreases in Glu are consistent with previous animal studies reporting decreased Glu concentrations in other brain regions such as anterior cingulate cortex, hippocampus and PFC of morphine-treated animals (Guo et al., 2005; Hao et al., 2005; Gao et al., 2007; Dehkordi et al., 2025).

NAA, a marker for neural viability, osmoregulation and energy metabolism (Taylor et al., 1995; Tallan et al., 1956; Moffett et al., 2007) responded differently to morphine in the wild-type and the GIRK2^+^/ ^-^ mutant mice. While the concentrations of NAA decreased after morphine in the wild-type mice, this same metabolite increased after morphine in the GIRK2^+^/ ^-^ mice. NAA has been used as a diagnostic marker for diseases and disorders of the CNS including brain ischemia, multiple sclerosis, Alzheimer disease and Canavan disease (Chen et al., 2000; Miller et al., 2003; Namboodiri et al., 2006; Mazibuko et al., 2020). However, with the exception of Canavan disease, most of these diseases have reported a decrease in the concentration of NAA. In Canavan disease, the increase in NAA is due to the absence of the enzyme aspartoacylase which is responsible for breaking down NAA (Moffet et al., 2007). Since many of the functions of NAA in the CNS are not fully understood, the significance of an increase in NAA after morphine in the GIRK2^+^/ ^-^ mutant mice is not clear. Whether this GIRK2^+^/ ^-^ mutation interferes with the enzymatic breakdown of NAA is not known.

NAAG, the most highly concentrated dipeptide in the brain, is usually measured in combination with NAA in ^1^H MRS due to large superposition of their spectra. NAAG can contribute 10–20 % of the signal that is often attributed to NAA (Pouwels and Frahm, 1997; Baslow and Guilfoyle, 2007). The release of NAAG is usually triggered by intense neuronal activation (Benarroch, 2008). Thus, assuming the decreased concentration of combined NAA + NAAG at the VRG in both groups of mice is due to the decrease in NAAG, this decrement most likely reflects morphine-induced decreased neuronal activity at this site. Altered levels of the antioxidant metabolite Gsh seen in transgenic mice is likely a compensatory response to morphine-induced oxidative stress (Averill-Bates, 2023).

### 4.1 Limitations

Although the homozygous GIRK2^−/−^ knockout strain might have more effectively abolished the GIRK2 channel activity, the high susceptibility of GIRK2^−^/^−^ animals to seizure activity (Signorini et al., 1997) would likely have confounded the results by impacting the metabolite profile in our region of interest. Another limitation for the interpretation of these results was that there were changes associated with the placebo pellet treatments. Such changes are likely caused by experimental stress (Gapp et al., 2017) which is thought to be minimized in the morphine treated animals due to morphine's anti-nociceptive and anti-stress properties (Bryant et al., 2009; Bali et al., 2015). The signal to noise ratio of the acquired ^1^H NMR spectra was a major concern when designing the study, given the small size and shape of the target brain region. Thus, the size of the voxel (volume of interest) was selected to optimize the signal to noise ratio, to maximize the coverage of target brain region (VRG) and to minimize contributions of extraneous tissues. However, the possibility that tissue from neighboring brain regions could contribute a small portion of the signal cannot be eliminated. The signal to noise ratio associated with small voxel size also makes identification and separation of particular signals much more difficult. As a result, concentrations of metabolites with overlapping spectra are summated and reported as total. This makes evaluation of morphine-induced changes of individual metabolites more challenging.

## 5 Conclusion

To our knowledge, this is the first *in vivo* ^1^H MRS study to quantify a broad range of metabolites in the VRG of both wild-type and GIRK2^+^/^−^ mutant mice, and to assess their response to prolonged morphine administration. The data demonstrate that the heterozygous GIRK2^+^/ ^-^ mutation alters baseline levels of metabolites in the VRG, notably affecting metabolites such as Glu, Ins, NAA + NAAG, and Glu + Gln. Despite these baseline differences, many of the morphine-induced metabolite changes were similar in the wild-type and GIRK2^+^/^−^ mice. This suggests that residual GIRK2 channel activity remains functional in the heterozygous mutants, or that alternative pathways such as KCNQ channels or μ-opioid receptor-independent mechanisms may mediate morphine's metabolic effects in this region. Together, these findings enhance our understanding of the neurochemical basis of opioid action in brainstem respiratory centers and underscore the multifaceted role of GIRK2 in shaping both baseline and opioid-responsive metabolic states.

## CRediT authorship contribution statement

**Ozra Dehkordi:** Conceptualization, Funding acquisition, Project administration, Investigation, Writing – original draft. **Stephen Lin:** Data curation, Investigation, Methodology, Resources, Software, Validation, Formal analysis. **Safia Mohamud:** Writing – review & editing. **Richard M. Millis:** Writing – review & editing. **Paul C. Wang:** Conceptualization, Funding acquisition, Project administration, Methodology, Resources, Supervision, Validation, Writing – review & editing.

## Funding

This project was supported (in part) by the 10.13039/100006545National Institute on Minority Health and Health Disparities of the 10.13039/100000002National Institutes of Health under Award Number 2U54MD007597. The content is solely the responsibility of the authors and does not necessarily represent the official views of the National Institutes of Health.

## Declaration of competing interest

The authors declare the following financial interests/personal relationships which may be considered as potential competing interests:

Ozra Dehkordi reports financial support, article publishing charges, equipment, drugs, or supplies, and travel were provided by 10.13039/100000002National Institutes of Health. If there are other authors, they declare that they have no known competing financial interests or personal relationships that could have appeared to influence the work reported in this paper.

## Data Availability

Data will be made available on request.
